# Congenital mucocele of the tongue: an unusually large presentation without obstruction

**DOI:** 10.11604/pamj.2023.44.100.38642

**Published:** 2023-02-20

**Authors:** Emmanuel Choffor-Nchinda, Vincent Verla Siysi

**Affiliations:** 1 Department of Surgery and Specialties, Faculty of Health Sciences, University of Buea, Buea, Cameroon,; 2 Surgical Unit, Buea Regional Hospital, Buea, Cameroon,; 3 Department of Internal Medicine and Paediatrics, Faculty of Health Sciences, University of Buea, Buea, Cameroon

**Keywords:** Congenital mucocele, lingual cyst, tongue, Cameroon

## Image in medicine

Congenital lingual mucoceles are rare tumours that present in variable sizes and locations within the tongue. We report the case of an infant whom we received at two weeks for a mass protruding from the oral cavity. On physical examination, the neonate had a good general condition. There was no respiratory or feeding difficulty. There was a large mucosa-covered, pink, soft tissue oral mass, occupying and expanding the oral fissure. The mass extended about 5 cm anterior to the fissure, and the lingual dorsum was identifiable intra-orally at its superior left surface. Head and neck computerized tomography scan showed an intra-lingual cystic mass, with mass effect on surrounding structures and no bone erosion. The diagnosis of a congenital mucocele of the tongue was made. Surgical excision was planned at six months, with very close monitoring for feeding or breathing abnormalities. The procedure consisted of a lateral right mucosal incision along the edge of the tongue, followed by meticulous blunt dissection and separation of the cyst from the surrounding tissues. The cyst was excised completely. It had an approximate volume of 180 ml. The mucosa was closed by interrupted stitches. The procedure was well tolerated and post-operative outcome was good. The patient was addressed to the odontostomatologist for management and eventual correction of occlusal abnormalities. Diligent assessment and management of the airway shared by the surgeon and anaesthetist was capital.

**Figure 1:**
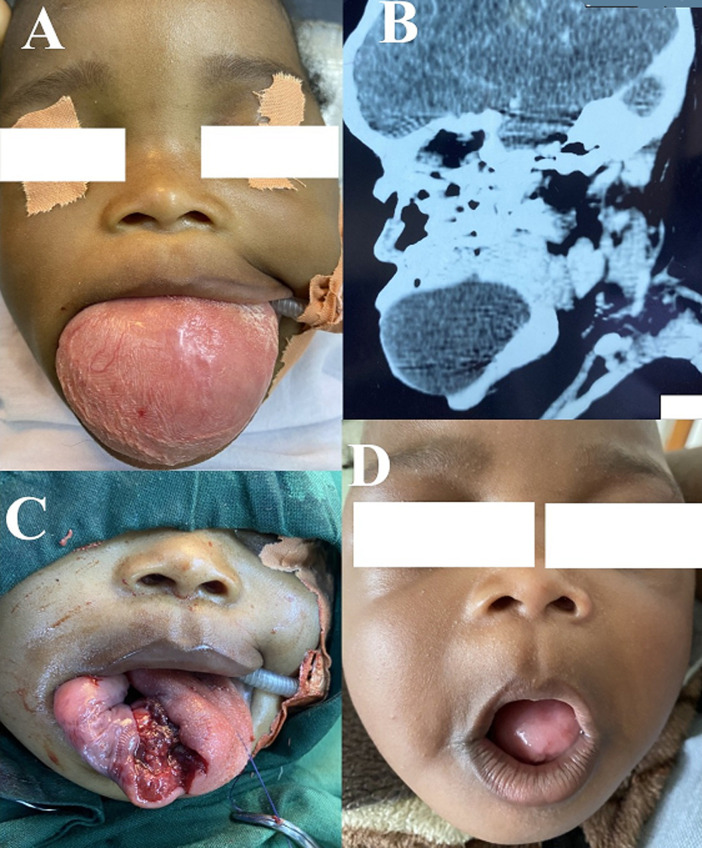
A) pre-operative aspect of mass; B) sagittal computed tomography (CT) scan showing intra-lingual cystic lesion; C) intra-operative view after excision; D) three weeks post-operative

